# The dose–response effects of arachidonic acid on primary human skeletal myoblasts and myotubes

**DOI:** 10.1080/15502783.2022.2164209

**Published:** 2023-01-03

**Authors:** Brandon M. Roberts, Alexander L. Kolb, Alyssa V. Geddis, Marshall A. Naimo, Ronald W. Matheny

**Affiliations:** Military Performance Division, US Army Research Institute of Environmental Medicine, Natick, MA, USA

**Keywords:** Cyclooxygenase, myogenesis, anabolic signaling, skeletal muscle

## Abstract

**Background:**

Cellular inflammatory response, mediated by arachidonic acid (AA) and cyclooxygenase, is a highly regulated process that leads to the repair of damaged tissue. Recent studies on murine C2C12 cells have demonstrated that AA supplementation leads to myotube hypertrophy. However, AA has not been tested on primary human muscle cells. Therefore, the purpose of this study was to determine whether AA supplementation has similar effects on human muscle cells.

**Methods:**

Proliferating and differentiating human myoblasts were exposed to AA in a dose-dependent manner (50–0.80 µM) for 48 (myoblasts) or 72 (myotubes) hours. Cell viability was tested using a 3-(4,5-Dimethylthiazol-2-Yl)-2,5-Diphenyltetrazolium Bromide (MTT) assay and cell counting; myotube area was determined by immunocytochemistry and confocal microscopy; and anabolic signaling pathways were evaluated by western blot and RT-PCR.

**Results:**

Our data show that the treatment of primary human myoblasts treated with 50 µM and 25 µM of AA led to the release of PGE_2_ and PGF_2α_ at levels higher than those of control-treated cells (*p* < 0.001 for all concentrations). Additionally, 50 µM and 25 µM of AA suppressed myoblast proliferation, myotube area, and myotube fusion. Anabolic signaling indicated reductions in total and phosphorylated TSC2, AKT, S6, and 4EBP1 in myoblasts at 50 µM of AA (*p* < 0.01 for all), but not in myotubes. These changes were not affected by COX-2 inhibition with celecoxib.

**Conclusion:**

Together, our data demonstrate that high concentrations of AA inhibit myoblast proliferation, myotube fusion, and myotube hypertrophy, thus revealing potential deleterious effects of AA on human skeletal muscle cell health and viability.

## 1. Introduction

Arachidonic acid (AA) is a fatty acid found in structural phospholipids of cell membranes. AA is present in low concentrations in circulation and can be cleaved from the cell membrane and metabolized via different enzymatic pathways [1]. The cyclooxygenase (COX) pathway, which is activated in response to cytokines or growth factors, metabolizes AA into prostanoids, including prostaglandin E_2_ (PGE_2_) and prostaglandin F_2α_ (PGF_2α_) [2,3]. Prostaglandins are mediators of inflammation, and play critical roles in skeletal muscle growth and development by influencing myoblast proliferation, differentiation, and fusion [4].

Previously, a study by Markworth et al. found that C2C12 skeletal muscle cells were affected in a dose-dependent manner by the treatment of AA [5]. High concentrations (50–100 µM) of AA resulted in myoblast death, while low concentrations, specifically, those under 25 µM AA, resulted in myotube hypertrophy. The results of these experiments suggest that high levels of inflammation may be detrimental, yet low levels of inflammation may exhibit positive effects on skeletal muscle growth. C2C12 cells are often used to study skeletal muscle at the cellular level, but several studies have revealed notable differences in glucose uptake, response to dexamethasone and glucocorticoids, mechanisms of insulin resistance, as well as the metabolism and transcriptome compared to other cell types [6–8]. Consequently, this may hinder the direct translation of these findings to human primary cell types. This may explain the equivocal results found in studies supplementing with AA to induce hypertrophy [9–12].

Given the potential differences between C2C12 cells and human primary cells, the aim of this study was to identify the effects of AA on proliferating and differentiating human skeletal muscle myoblasts in a dose-dependent manner. We hypothesized that AA would result in dose-dependent myotube growth and fusion in primary human myotubes up to 25 µM of AA and that 50 µM of AA would result in myoblast death.

## 2. Methods

### Materials and reagents

#### Cell culture and arachidonic acid

Human Skeletal Myoblasts (Thermo Fisher Scientific) were grown and expanded in Skeletal Muscle Cell Growth Media and Bullet Kit at 37°C and 5% CO_2_ for all experiments on myoblasts. Human Skeletal Myotubes were differentiated from myoblasts (Lonza Technologies) using low-glucose Dulbecco’s Modified Eagle’s Medium supplemented with 2% horse serum at 37°C and 5% CO_2._ All experiments were performed within six cell passages of receipt from the vendor. Cells for proliferative experiments were grown and then seeded in six-well dishes for RNA/protein and 12-well dishes for immunofluorescence at 1.4 × 10^4^ cells/cm^2^. The media was refreshed every 24 hours. For differentiation experiments, the cells were seeded at 3.8 × 10^6^ cells/cm^2^ and then differentiated in low-glucose Dulbecco’s Modified Eagle’s Medium with 2% horse serum at 37°C and 5% CO_2_. AA was stored at −20°C upon arrival and diluted in ethanol and cells were exposed, where appropriate, to various doses at a final concentration of 0.1% (Item No. 90010, Cayman Chemicals, Ann Arbor, MI). In all cases, control cells were treated at equivalent concentrations of ethanol. For all experiments, media and AA were replaced every 24 hours. Myoblast experiments lasted 48 hours and myotube experiments lasted 72 hours.

#### ELISAs

Conditioned cell media or protein lysates were collected and prepared for enzyme-linked immunosorbent assay (ELISA) as previously described [13,14]. ELISAs were used to measure prostaglandin hormone concentrations PGE_2_ and PGF_2α_ from conditioned media.

#### Viability assays

Cell viability was measured using 3-(4,5-Dimethylthiazol-2-Yl)-2,5-Diphenyltetrazolium Bromide (MTT) assay and cell counting. Human skeletal muscle myoblasts were allowed to grow for 48 hours and then the MTT assay was performed according to the manufacturer’s instructions [13,14]. Cell counting was used to assess live and dead cells using an automated cell counter. Myoblasts were seeded and grown for 48 hours in respective conditions. Afterward, myoblasts were trypsinized, neutralized, and stained with Trypan Blue (0.4%) to stain for live cells. Cells were loaded into countess cell counting chamber slides and counted on an Invitrogen Countess Automated Cell Counter. Live and dead cells were counted.

#### Protein extraction and immunoblotting

Cellular protein extraction, Bradford analysis, and immune blotting were conducted as previously described [13,14]. Purified COX1 or COX2 peptide proteins were used as positive controls. Densiometric quantification analysis was done using NIH ImageJ 1.60 [15]. All images representative images are from the same set of cells, so vinculin staining is repeated on multiple figures for comparison. Antibodies are listed in Table 1.

**Table 1.** Materials and reagents information.

Material/Reagent	Company	Catalog #
Human Skeletal Myoblasts	Lonza Technologies (Portsmouth, NH, USA)	2580
Skeletal Muscle Growth Media and Bullet Kit		3245
Trypsin and Trypsin Neutralizing Solution Reagent Pack		5034
MTT Proliferation Assay	ATCC (Manassas, VA, USA)	30-1010K
Human Skeletal Myoblasts	Thermo Fisher Scientific (Waltham, MA, USA)	A11440 & A12555
Low-Glucose Dulbecco’s Modified Eagle’s Medium		11885092
Horse Serum		26050070
ptgs1		Hs00377726_m1
ptgs2		Hs00153133_m1
elf1		Hs00152844_m1
myh1		Hs00428600_m1
myh2		Hs00430042
myh3		Hs01074230
myh7		Hs01110632
myh8		Hs00267293
Vinculin	Cell Signaling Technologies Antibodies (Danvers, MA, USA)	13901
p-p70 T389		9234
Total p70		2708
p-AKT S473		9271
Total AKT		9272
p-S6 S235/236		4858
p-S6 S240/244		2215
Total S6		2217
COX1		9896
COX2		12282
p-TSC1 S1387		23402
Total TSC2		4308

#### RNA isolation, cDNA synthesis, and RT-PCR

RNA isolation, cDNA synthesis, and RT-PCR were performed as previously described by Matheny et al. [13,14], Briefly, cDNA was synthesized using a high-capacity cDNA reverse transcription kit with RNase inhibitor (catalog number 4374966; Life Technologies) and 2 μg RNA in a 40-μl final reaction volume according to the manufacturer’s instructions. cDNA was either used immediately or stored at −80°C until use. RT-PCR was performed using 20 ng cDNA with the parameters set as follows: 10 min at 95°C, followed by 40 cycles of 95°C for 15 seconds and 60°C for 60 seconds. Measurements were taken in the exponential phase when fluorescence exceeded the threshold of detection above the background (threshold cycle [CT] values). Measurements of gene expression for all transcripts were taken when the threshold of detection exceeded background (CT value) and was calculated using the −2^ΔΔCT^ method and normalized to the level of *elf1* gene expression. PCR probes are listed in Table 1.

#### Immunocytochemistry, microscopy, and image analysis

Cells were seeded and differentiated on collagen‐coated coverslips as previously described [14,16] and then stained with MF 20, which recognizes all forms of MyHC, from the Developmental Studies Hybridoma Bank. Images were captured using a Zeiss LSM700 confocal microscope and Zen analysis software. Images were derived from five randomly captured fields for each treatment group. Myotube fusion index was determined by counting the nuclei in every myotube (defined as MyHC‐positive cells containing ≥2 nuclei) per field and dividing by the total number of nuclei in the field. Images were analyzed using ImageJ, which was downloaded from the National Institutes of Health website (Bethesda, MD, USA) [15]. Results are presented as means ± standard errors from three independent experiments.

#### Statistics

Data are presented as mean ± SEM. Statistical analyses were performed using a Student’s t-test or one-way analysis of variance (ANOVA) with a Dunnett’s test post-hoc, as appropriate. A *p*-value of <0.05 was considered significant. N = 3–4 independent experiments.

## 3. Results

### Arachidonic acid stimulates PGE_2_ and PGF_2α_ in primary human myoblasts in a dose-dependent fashion

**Figure 1.**
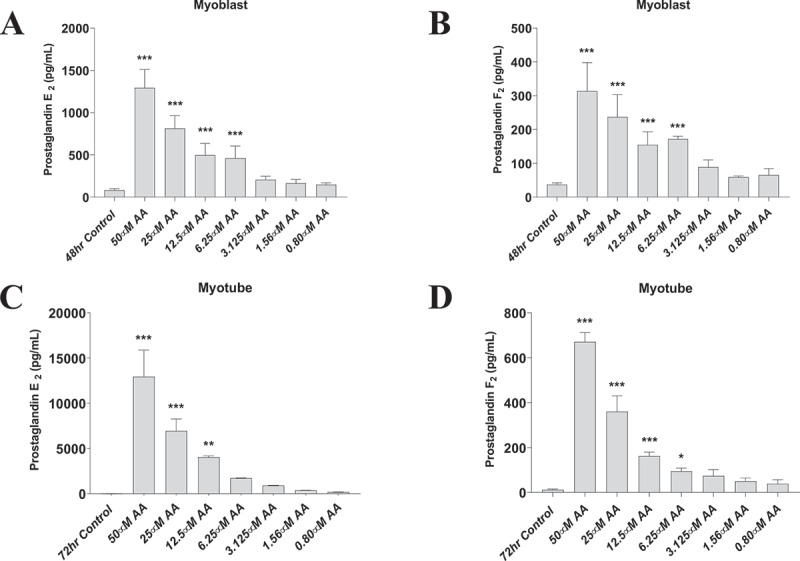
AA stimulates prostaglandin production in a dose-dependent manner. Prostaglandin hormones PGE_2_ and PGF_2α_ were measured using ELISAs. (A) PGE_2_ and (B) PGF_2α_ concentrations are shown for proliferating myoblasts treated with AA for 72 hours. (C) PGE_2_ and (D) PGF_2α_ concentrations for differentiating myotubes treated with AA for 48 hours. Data are expressed as mean ± SEM (***p < 0.001; **p < 0.01; *p < 0.05; all compared to respective control).

AA release stimulates the formation of prostaglandins which mediate the cellular response to inflammation. We therefore examined the effects of AA on both primary human myoblasts and myotubes on prostaglandin formation. Provision of various concentrations of AA dose-dependently increased both PGE_2_ and PGF_2α_ concentrations in primary human myoblasts and myotubes (Figure 1A-D), demonstrating the efficacy of AA supplementation in this model system.

### Arachidonic acid leads to a reduction in primary human myoblast proliferation, growth, and maturation

**Figure 2.**
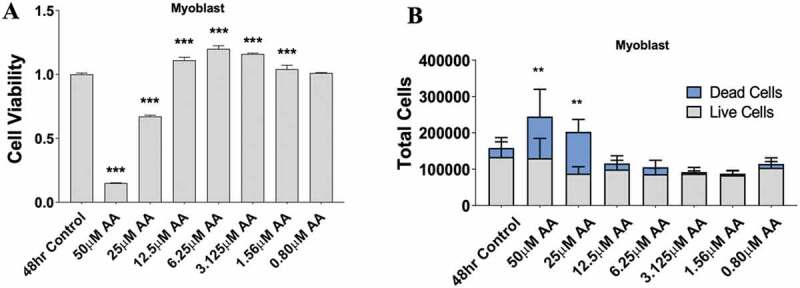
AA reduces myoblast viability at high and moderate doses. Skeletal muscle myoblasts were proliferated for 48 hours with various concentrations of AA. (A) Cell viability was measured using 3-(4, 5-Dimethylthiazol-2-Yl)-2, 5-Diphenyltetrazolium Bromide assay. Viability was affected at concentrations of 50, 25, 12.5, 6.25, 3.15, and 1.56 µM AA. (B) Cell counts were measured using Trypan Blue (0.4%) and an automated counter. Data are expressed as mean ± SEM (***p < 0.001; **p < 0.01; *p < 0.05; all compared to control). n = 3 independent experiments.

**Figure 3.**
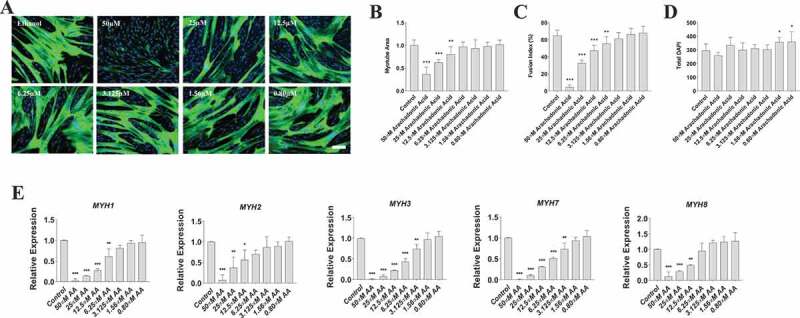
AA reduces myotube formation in a dose-dependent manner. Differentiating skeletal muscle myoblasts were treated with decreasing concentrations of AA for 72 hours and then fixed and prepared for fluorescent confocal microscopy and gene using an antibody that recognizes embryonic myosin heavy chain. (A) Representative images were generated using a Zeiss LSM 700 using Zen software. AA concentrations are provided for each image. Analyses of images from two independent experiments for (B) myotube area, (C) myotube fusion percentage, and (D) total DAPI. Myotubes were also analyzed for myosin heavy chain mRNA expression of (E) MYH1, MYH2, MYH3, MYH7, and MYH8. Data are expressed as mean ± SEM (***p < 0.001; **p < 0.01; *p < 0.05; all compared to control). n = 3 independent experiments.

Since AA demonstrated a clear influence on prostaglandin production, we investigated the dose-dependent effects of AA on myoblast proliferation, myotube area, and myotube fusion to determine its effect on myogenesis. Myoblasts were treated for 48 hours during proliferation. We found that high concentrations of AA (50 µM and 25 µM) reduced myoblast viability by 85% and 32%, respectively (*p* < 0.001) (Figure 2A) and increased the number of dead cells (Figure 2B). On the other hand, low concentrations (12.5–1.56 µM AA) caused increased myoblast proliferation (*p* < 0.001) (Figure 2). Next, we examined the influence of AA on changes in myotube area and fusion indices (Figure 3A). We found 50 µM of AA reduced myotube area by 63% (*p* < 0.001), 25 µM reduced myotube area by 38% (*p* < 0.001), and 12.5 µM decreased myotube area by 23% (*p* < 0.01) (Figure 3B). Myotube fusion was reduced by 93% with 50 µM AA (*p* < 0.001), by 50% with 25 µM AA, by 29% with 12.5 µM AA (*p* < 0.001), and by 14% with 6.25 µM AA (*p* < 0.01) (Figures 3A–3C). When measuring total myonuclei, numbers increased 14% and 15% at concentrations of 1.56 and 0.80 µM AA, respectively (*p* < 0.01) (Figure 3D).

To determine whether these AA-induced changes in myotube area were associated with decreased myosin heavy chain (MYH) gene expression, we measured mRNA levels of MYH1, MYH2, MYH3, MYH7, and MYH8 that constitute myosin fast, slow, embryonic, and perinatal species in human skeletal muscle (Figure 3E). We found that AA decreased mRNA expression of MYH1 by 99% at 50 µM (*p* < 0.001), 90% at 25 µM (*p* < 0.001), 80% at 12.5 µM (*p* < 0.001), and 40% at 6.25 µM (*p* < 0.01). We found that AA decreased mRNA expression of MYH2 by 90% at 50 µM (*p* < 0.001), 70% at 25 µM (*p* < 0.01), and 45% at 12.5 µM (*p* < .05). MYH3 decreased by AA by 99% at 50 µM (*p* < 0.001), 90% at 25 µM (*p* < 0.001), 76% at 12.5 µM (*p* < 0.001), 60% at 6.25 µM (*p* < 0.001), and 30% at 3.125 µM (*p* < 0.01). We found similar results with MYH7 and MYH8. Specifically, MYH7 decreased by AA by 99% at 50 µM (*p* < 0.001), 90% at 25 µM (*p* < 0.001), 70% at 12.5 µM (*p* < 0.001), 50% at 6.25 µM (*p* < 0.001), and 25% with 3.125 µM AA (*p* < 0.01). Likewise, MYH8 decreased 90% at 50 µM (*p* < 0.001), 70% at 25 um µM AA (*p* < 0.01), and 62% at 12.5 µM AA (*p* < 0.01). Collectively, these data reveal a dose-dependent effect of AA on myosin gene expression, myotube fusion, and myotube area.

### High level of arachidonic acid impairs protein synthetic pathways in human myoblasts

The proliferation, growth, and maturation of primary human myoblasts are heavily dependent on protein kinase B (AKT) signaling. Therefore, we examined the dose-dependent effects of 48 hours of AA treatment on the AKT signaling pathway in primary human myoblasts.

**Figure 4.**
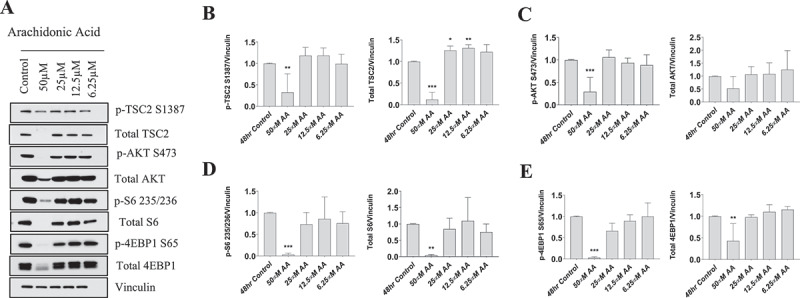
High doses of AA impair the protein kinase B/AKT pathway in proliferating human skeletal myoblasts. Western immunoblotting was performed using lysates from human skeletal myoblasts that were treated for 48 hours with AA. (A) Representative images are shown. Densiometric quantification was used and proteins were normalized to their respective totals for (B) p-TSC2 S138, (C) p-AKT S473, (D) p-S6 235, and (E) p-4EBP1 S65. Data are expressed as mean ± SEM and represent four independent experiments (***p < 0.001; **p < 0.01; *p < 0.05; all compared to control). n = 4 independent experiments.

We observed that phosphorylated tuberous sclerosis complex 2 (TSC2) and Total TSC2 were decreased with 50 µM AA when normalized to vinculin (*p* < 0.01, *p* < 0.001) (Figure 4B). TSC2 was slightly elevated at 25 µM and 12.5 µM when normalized to vinculin (*p* < 0.05, *p* < 0.01) (Figure 4). When normalizing the abundances of p-TSC2 S1387 to Total TSC2, p-TSC2 S1387/Total TSC2 was elevated fourfold with 50 µM AA, but did not change with any other dose of AA (Supplemental Figure 1).

Phosphorylated AKT at S473 decreased at 50 µM AA when normalized to vinculin (*p* < 0.001), whereas total AKT abundance was not different when normalized to vinculin at any AA concentration (Figure 4C). No differences were observed across AA concentrations when p-AKT S473 was normalized to total AKT (Supplemental Figure 1).

Both phosphorylated ribosomal protein S6 S235/236 and Total S6 were decreased at 50 µM AA when normalized to vinculin (*p* < 0.001) (Figure 4D). No differences in p-S6 235/Total S6 were observed at any AA dose (Supplemental Figure 1).

Phosphorylated 4EBP1 and Total 4EBP1 were both decreased with 50 µM AA when normalized to vinculin (*p* < 0.001, *p* < 0.01) (Figure 4E). However, when normalized to total 4EBP1, p-4EBP1 was decreased 90% with 50 µM AA (*p* < 0.001) and 40% with 25 µM AA (*p* < 0.01) (Supplemental Figure 1).

### Arachidonic acid treatment does not affect COX-1 or COX-2 protein levels in myoblasts

**Figure 5.**
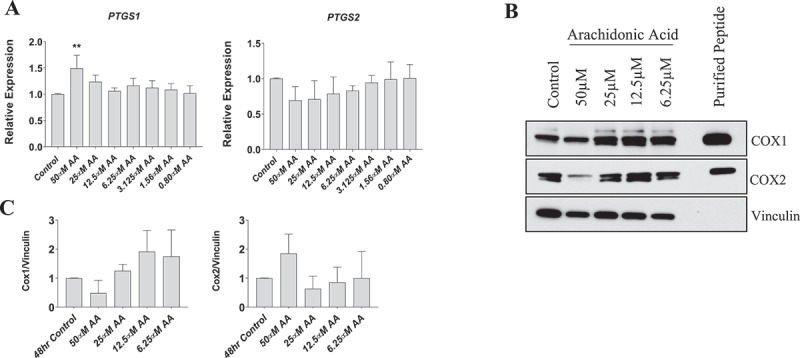
Effect of AA on COX inflammatory pathway in proliferating human skeletal myoblasts. (A) PTGS1 and PTGS2 mRNA levels were examined in cells treated with various concentrations of AA for 48 hours. COX protein levels were examined in cells treated with various concentrations of AA or equivalent diluent (ethanol) for 48 hours. (B) Representative western blots are shown; purified COX proteins were run to ensure accuracy of band identification. (C) Densiometric quantifications of Cox-1 and Cox-2. Data are expressed as mean ± SEM and represent three independent experiments (***p < 0.001; **p < 0.01 *p < 0.05; all compared to control).

Cyclooxygenase enzymes mediate the conversion of AA to prostaglandins, which were associated with reduced myoblast proliferation and differentiation (Figures 2 and 3). To determine whether these AA-mediated effects were related to changes in COX abundance, we examined the gene and protein expression of COX1 and COX2 in myoblasts. Gene expression of COX-1 (*PTGS1*) increased by ~50% in myoblasts treated with 50 µM AA (*p* < 0.01) (Figure 5A). No differences were observed in COX-2 (*PTGS2*) gene expression at any AA concentration. Despite the increased expression of *PTGS1* mRNA in myoblasts treated with 50 µM AA, there were no differences in COX1 or COX2 protein levels when normalized to vinculin (Figures 5B and 5C).

### Protein synthetic pathways are unperturbed following AA treatment in myotubes, despite AA-induced increase in COX-2 protein

**Figure 6.**
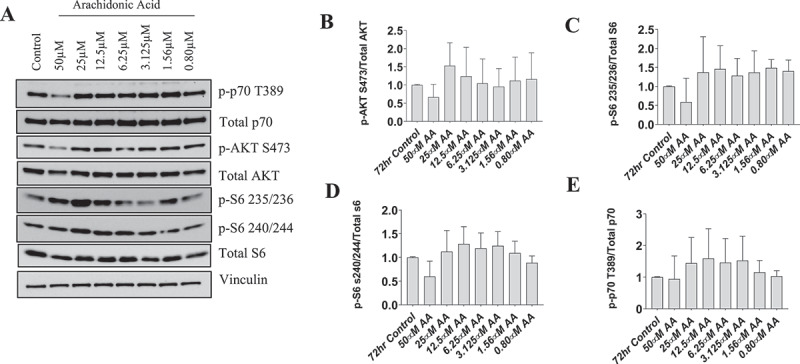
AA does not impair the protein kinase B/AKT pathway in differentiating human skeletal myotubes. Various concentrations of AA were used to treat human skeletal myotubes for 72 hours. (A) Representative western blots are shown. Densitometric quantification was used and proteins were normalized to their respective totals for (B) p-AKT, (C) p-S6 235, (D) p-S6 240, and (E) p-p70 T389. Data are expressed as mean ± SEM and represent four independent experiments (***p < 0.001; **p < 0.01 *p < 0.05).

To determine whether protein synthetic pathways in myotubes were altered to a similar degree as observed in myoblasts, we analyzed the dose-dependent effects of AA on these pathways in human myotubes treated for 72 hours during differentiation with AA. In contrast to our findings in myoblasts, treatment of myotubes with any AA concentration did not reduce the abundance of total or phosphorylated p70S6K, AKT, or S6 (Figures 6A–6B).

**Figure 7.**
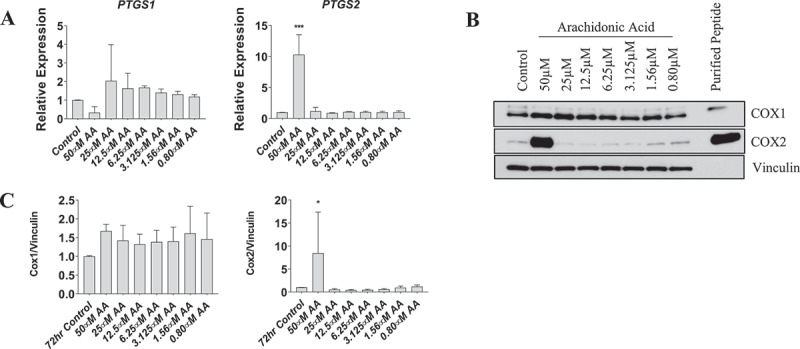
Effect of AA on COX inflammatory pathway in differentiating human skeletal myotubes. (A) PTGS1 and PTGS2 mRNA levels were examined in cells treated with various concentrations of AA for 48 hours. COX protein levels were examined in cells treated with various concentrations of AA or equivalent diluent (ethanol) for 48 hours. (B) Representative western blots are shown; purified COX proteins were run to ensure accuracy of band identification. (C) Cox1 and Cox2 protein levels in cells treated with various concentrations of AA for 48 hours. Data are expressed as mean ± SEM and represent three independent experiments (***p < 0.001; **p < 0.01 *p < 0.05; compared to control).

We next examined the gene expression and protein levels of COX-1 and COX-2 in myotubes. Expression of *PTGS1* was not affected by AA at any concentration; however, expression of *PTGS2* increased ~10-fold following treatment with 50 µM AA (*p* < 0.01) (Figure 7A). COX-2 protein was increased ~8-fold following 50 µM AA (*p* < 0.01), whereas COX-1 protein was unaffected following AA administration at any concentration (Figures 7b–c).

### Celecoxib treatment does not attenuate the effects of arachidonic acid at high doses

**Figure 8.**
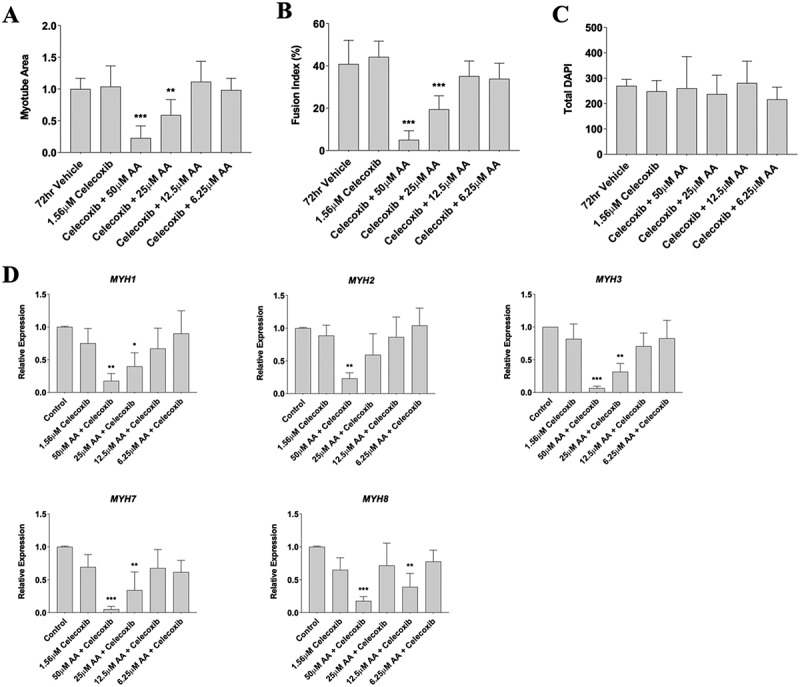
Celecoxib does not attenuate the effect of AA at high doses on myotubes. Differentiating skeletal muscle myoblasts were treated with decreasing concentrations of AA and Celecoxib for 72 hours and then fixed and prepared for fluorescent confocal microscopy and gene using an antibody that recognizes embryonic myosin heavy chain. Analyses of images experiments for (A) myotube area, (B) myotube fusion percentage, and (C) total DAPI. Myotubes were also analyzed for myosin heavy chain mRNA expression of (D) MYH1, MYH2, MYH3, MYH7, and MYH8. Data are expressed as mean ± SEM (***p < 0.001; **p < 0.01; *p < 0.05; all compared to control). n = 3 independent experiments.

In a final experiment, we used a strong COX-2 inhibitor (Celecoxib) to determine whether AA was acting through the COX-2 pathway. Given the previously mentioned deleterious effect of AA on myotube size, fusion, and myosin heavy chain genes, we utilized myotubes for these experiments. Furthermore, based on previous experiments with Celecoxib and AA, we chose 1.56 µM Celecoxib (Matheny et al., in press) and tested dosages of AA ranging from 50 to 6.25 µM. We found that 50 µM of AA and 1.56 µM Celecoxib reduced myotube area by 85% (*p* < 0.001) and 25 µM of AA and 1.56 µM Celecoxib reduced myotube area by 45% (*p* < 0.01) (Figure 8A). Myotube fusion demonstrated similar results, with an 80% decrease in fusion index with 50 µM of AA and 1.56 µM Celecoxib (*p* < 0.001) and a 60% decrease with 25 µM of AA and 1.56 µM Celecoxib (*p* < 0.001) (Figure 8B). No changes were found in total DAPI (Figure 8C). When analyzing mRNA expression, we found that 50 µM of AA and 1.56 µM Celecoxib reduced MYH1 by 82% (*p* < 0.01), MYH2 by 75% (*p* < 0.01), MYH3 by 90% (*p* < 0.001), MYH7 by 92% (*p* < 0.001), and MYH8 by 78% (*p* < 0.01). Similarly, 25 µM of AA and 1.56 µM Celecoxib reduced MYH1 by 60% (*p* < 0.05), MYH3 by 65% (*p* < 0.01), and MYH7 by 71% (*p* < 0.01) (Figure 8D). Collectively, these data reveal that Celecoxib does not influence the effect of AA on myosin gene expression, myotube fusion, and myotube area at high doses (50–25 µM) but may attenuate deleterious effects at moderate doses (12.5–6.25 µM).

## 4. Discussion

The purpose of this study was to identify the effects of AA on proliferating and differentiating human skeletal muscle myoblasts and myotubes in a dose–response manner. Our results demonstrate that prostaglandins PGE_2_ and PGF_2α_ are elevated at high and moderate doses of AA (50–6.25 µM) but not at low doses (3.125–0.80 µM). Furthermore, high and moderate doses of AA resulted in reduced myotube area and myotube fusion; yet, they did not affect myonuclear number. This finding was associated with a dose-dependent decrease in the expression of myosin heavy chain isoforms, where higher doses of AA resulted in decreased *MYH1, MYH2, MHY3, MHY7*, and *MHY8* transcription, whereas lower doses had no effect. Surprisingly, analysis of signaling molecules involved in protein synthesis showed no significant changes in myotubes, whereas levels of both phosphorylated and total proteins were significantly reduced in myoblasts treated with 50 µM AA. The use of a strong COX-2 inhibitor, Celecoxib, did not influence deleterious effects of high doses of AA, which hints at another mechanism independent of the COX pathway. Taken together, these data reveal that high doses of AA inhibit myoblast proliferation, and that high and moderate concentrations of AA do not stimulate fusion or hypertrophy in human primary muscle cells.

Previous data using C2C12 murine muscle cells suggest that AA can induce a dose–response effect opposite to our findings, with myotube diameter and total protein increasing in parallel with AA concentration, up to 25 µM AA [5]. However, the study indicated that there were no changes in the percentage of differentiation or myotube fusion compared to a control [5]. In contrast, in human myotubes, we found reduced myotube fusion at similar concentrations (including 25 µM AA) to those used previously [5]. Other research using a similar AA dose (20 µM) in C2C12 cells observed fewer differentiated myotubes, but more proliferative myoblasts, indicating a prolonged proliferation and delayed differentiation, which is in line with our results [17]. Similarly, others have found that 10 µM AA increases C2C12 proliferation [18]. Research using 10 µM AA in bovine muscle cells reported increases in myoblast proliferation, like our observations [19]. However, unlike our findings, they found increases in *MYOG* and *MYH3*, with no inhibitory effects on myotube fusion [19]. In another study, which used L6 skeletal muscle cells treated with 20 µM AA for 4 days during differentiation, myotube fusion was increased along with myosin heavy chain protein abundance [20].

The differences in findings between our studies and others could be due to several factors. Most notably, the cell lines were derived from different species (humans vs. mice or bovine), which could certainly influence the findings [6–8]. There were also subtle differences in methods used to calculate myotube area and fusion that could influence outcomes. It should be noted, however, that the morphology of differentiated myotubes can vary across species; to this end, methods are expected to differ to best accommodate the morphology of the species being assessed. Nevertheless, there remains the possibility that methodological differences may contribute to differences in findings. A third difference that may result in discrepancies across studies relates to the number of cells seeded onto plates or slides prior to the proliferation or fusion experiments as well as the time spent with AA treatment. It has long been recognized that skeletal muscle proliferation and differentiation are dependent on myoblast density, where conditions facilitating a high degree of cell-to-cell contact result in rapid and robust myotube formation, and conditions exhibiting only sparse densities result in only limited proliferation and differentiation capacity. Ultimately, our observations contradict the findings that AA induces muscle growth across the proliferation and differentiation spectrum. Moreover, our mechanistic data on the AKT pathway support our phenotypic findings in myotubes, revealing that high levels of AA inhibit proliferation and differentiation at high doses, rather than induce myotube hypertrophy.

An interesting finding from our experiments was the different physiological responses between myotubes and myoblasts. Namely, PGE_2_ was 10-fold higher and PGF_2α_ was 2-fold higher when treating myotubes with AA compared to myoblasts, at the same doses. This may explain the elevated COX-2 mRNA and protein levels observed at the highest AA concentration in myotubes, and may underlie, at least in part, the reduced fusion and myotube areas. These results are not in agreement with findings in C2C12 cells, where increasing amounts of prostanoids caused increases in myotube hypertrophy [21]. A potential explanation could be that the myoblasts are treated with media containing dexamethasone, and our myotube media does not contain dexamethasone. Indeed, our data reveal that growth media without dexamethasone treatment increases the amount of PGE_2_ and PGF_2α_ in myoblasts.

From a translational perspective, supplementation of AA in humans to promote muscle growth has been equivocal. For example, AA (1 g/day) did not result in greater gains in strength, muscle mass, or influence markers of muscle hypertrophy after 7 weeks of strength training compared to a placebo [12]. Other research with high doses of AA supplementation (1.5 g/day) found that AA can increase lean body mass and muscle power in strength-trained individuals compared to a placebo [9]. In a separate rodent model designed to mimic a strength-training stimulus, a human equivalent dose was given to rodents (44 mg/day) for 8 days before anabolic and catabolic signaling markers and muscle protein synthesis were measured after an acute exercise bout. The authors found AA supplementation did not enhance muscle protein synthesis or enhance selected mRNAs in rodents compared to the control condition (18). Along these lines, other research in humans indicates that 4 weeks of AA supplementation (1.5 g/day) does not alter the myofibrillar muscle protein synthesis response following an acute bout of resistance exercise. There was an expected effect of resistance exercise to stimulate translation initiation signaling via the mTOR pathway during the first 4 hours post-exercise; however, this response was also not influenced by AA supplementation [11]. These data further support our findings that AA does not activate the AKT/mTOR pathway and leads to increased muscle protein synthesis or muscle hypertrophy. However, our findings that supraphysiological doses of AA are detrimental to skeletal muscle are not parallel to any study in humans. The equivocal result across human studies indicates that there could be differential effects in the skeletal muscle response depending on the species, exercise protocols, AA dose, and timing of data collection that need to be studied.

There are some limitations to our study. First, we did not directly compare AA to an enzymatically deactivated structural analog of AA, such as eicosatetraenoic acid (ETYA), which can act as a nonspecific COX inhibitor by competing with AA for enzyme binding. The rationale for not using ETYA as a control is that physiological effects induced by AA have also been reported to be induced by ETYA [22,23]. Second, we did not explore all the potential mechanisms by which AA could influence proliferation and myotube fusion, which will be a focus of future research. One strength of our study is the data showing the transcriptional and protein data on COX-1 and COX-2, indicating only high doses of AA increase COX2 protein; these data and our recent findings indicating a COX-2 inhibitor (i.e. celecoxib), but not a COX-1 inhibitor (NS-398), is detrimental to skeletal muscle cells are the rationale for not testing a COX-1 inhibitor in tandem with AA [24]. It is unclear whether our results are due to the unique experimental conditions and cell lines used compared to others. We utilized media that includes dexamethasone, which is known to inhibit prostaglandin production by blocking phospholipase A2 in non-muscle cells [25–27], and the effects of dexamethasone at low doses during myogenesis are complex and not well understood [28,29]. However, dexamethasone was present under all treatment conditions in myoblasts, which permits confidence when comparing across experimental conditions. When completing additional experiments to determine the role of dexamethasone on prostaglandin production, we found that it reduced PGE2 in the media rather than increased it (Supplemental Figure 4). Finally, the strength of our design was the dose-response since it is unknown what this translates to in current human papers but covers a broad spectrum of doses.

In conclusion, our findings demonstrate that high concentrations of AA reduce primary human myoblast proliferation, and that high and moderate concentrations of AA reduce myoblast differentiation. Reduced proliferation may occur partially as a consequence of altered AKT pathway signaling, whereas alternate pathway(s) may regulate AA-mediated reductions in differentiation [30,31]. High concentrations of AA may also affect survival- and death-mediating pathways to favor cell death processes such as apoptosis or necrosis. Such actions may result from the blockade of vital survival signaling pathways or may result from the toxicity of AA itself. Finally, the responses of primary human myoblasts to high doses of AA supplementation are distinct from previous findings in murine C2C12 cells, but in line with others. Given the equivocal results in human, cell, and rodent studies, more research is needed to understand the effects of AA on skeletal muscle myogenesis and if it is beneficial for muscle growth. Ideally, these experiments would be completed in humans using an anabolic exercise program supplemented with AA with measurements of prostaglandin production, muscle protein signaling, and muscle inflammation signaling, using a randomized, controlled, dose–response design with acute and chronic outcomes.

## 5. Disclaimer

The opinions or assertions contained herein are the private views of the authors and are not to be construed as official or reflecting the views of the Army or the Department of Defense. Any citations of commercial organizations and trade names in this report do not constitute an official Department of the Army endorsement of approval of the products or services of these organizations. The authors have no conflict of interests to report.

## Supplementary Material

Supplemental Material

## Nonstandard abbreviations

4E-BP1 – Eukaryotic translation initiation factor 4E-binding protein 1

AA – Arachidonic acid

AKT – Protein kinase B, is a serine/threonine-specific protein kinase

COX-1 – Cyclooxygenase 1

COX-2 – Cyclooxygenase 2

DAPI – 4’,6-diamidino-2-phenylindole

DMSO – Dimethyl sulfoxide

ERK – Extracellular signal-regulated kinase

ELISA – Enzyme-linked immunosorbent assay

MTT – 3-(4,5-Dimethylthiazol-2-Yl)-2,5-Diphenyltetrazolium Bromide

mTOR – Mechanistic target of rapamycin

MYH – Myosin heavy chain

NSAID – Non-steroidal anti-inflammatory drug

p-70 – Ribosomal protein S6 kinase beta-1

p-s6 – Ribosomal S6 protein

PGE_2_ – Prostaglandin E_2_

PGF_2α_ – Prostaglandin F_2α_

PTGS2 – Prostaglandin-endoperoxide synthase 2

RT-PCR – Reverse transcription polymerase chain reaction

TSC2 – Tuberous sclerosis complex 2
