# Supplementary material for: The dose–response effects of arachidonic acid on primary human skeletal myoblasts and myotubes
Source: J Int Soc Sports Nutr. 2023 Jan 3;20(1):2164209. doi: 10.1080/15502783.2022.2164209 (PMC9817121; doi:10.1080/15502783.2022.2164209)
Supplement: Supplemental Material [file RSSN_A_2164209_SM0350.docx]

**Supplementary Figure 1.** High doses of Arachidonic Acid increase TSC2 phosphorylation and decrease 4EBP1 phosphorylation in myoblasts. Phosphorylated-TSC2 S1387/Total TSC2 was elevated 50 µM AA, but not changed with any other dose of AA. When normalized to total 4EBP1, p-4EBP1 was decreased with 50 µM AA and with 25 µM AA. No significant differences were observed across AA concentrations for p-AKT S473/Total AKT or p-S6 235/Total S6. Data is expressed as mean ± SEM and represents 3 independent experiments (***p<0.001; **p<0.01 *p<0.05; compared to control).

**
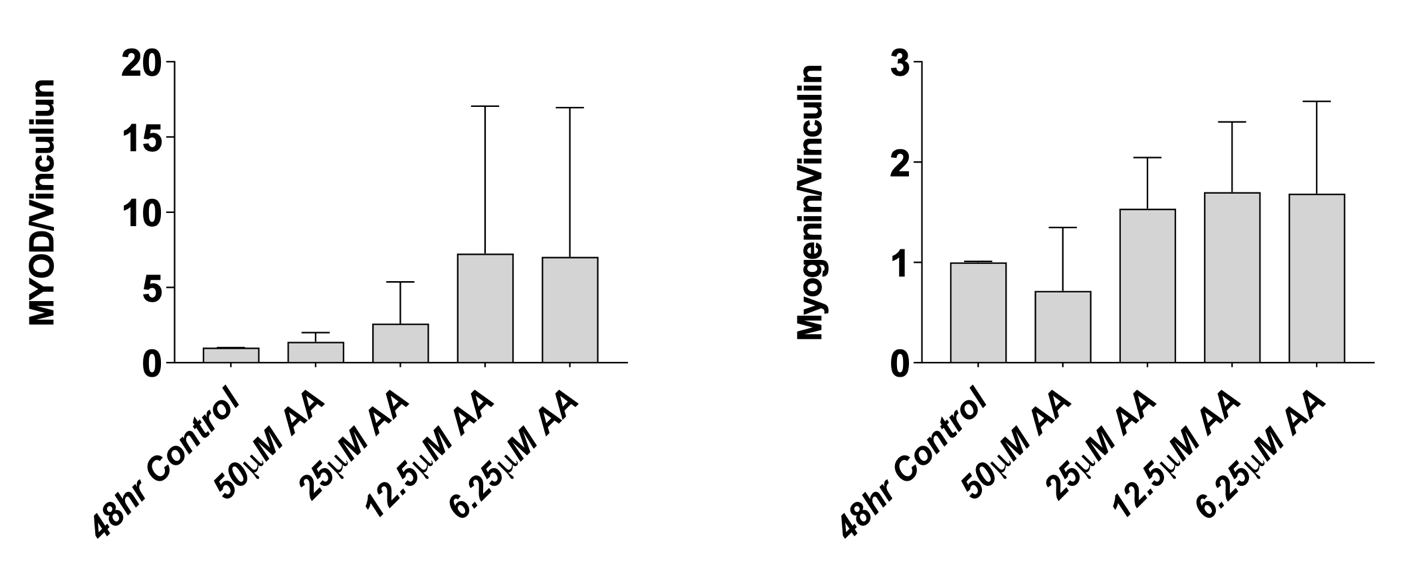
**

**Supplementary Figure 2.** Arachidonic acid does not influence myogenic regulatory factors. MyoD and Myogenin protein abundance were not changed with any AA treatment. Data is expressed as mean ± SEM and represents 3 independent experiments (***p<0.001; **p<0.01 *p<0.05; compared to control).

**
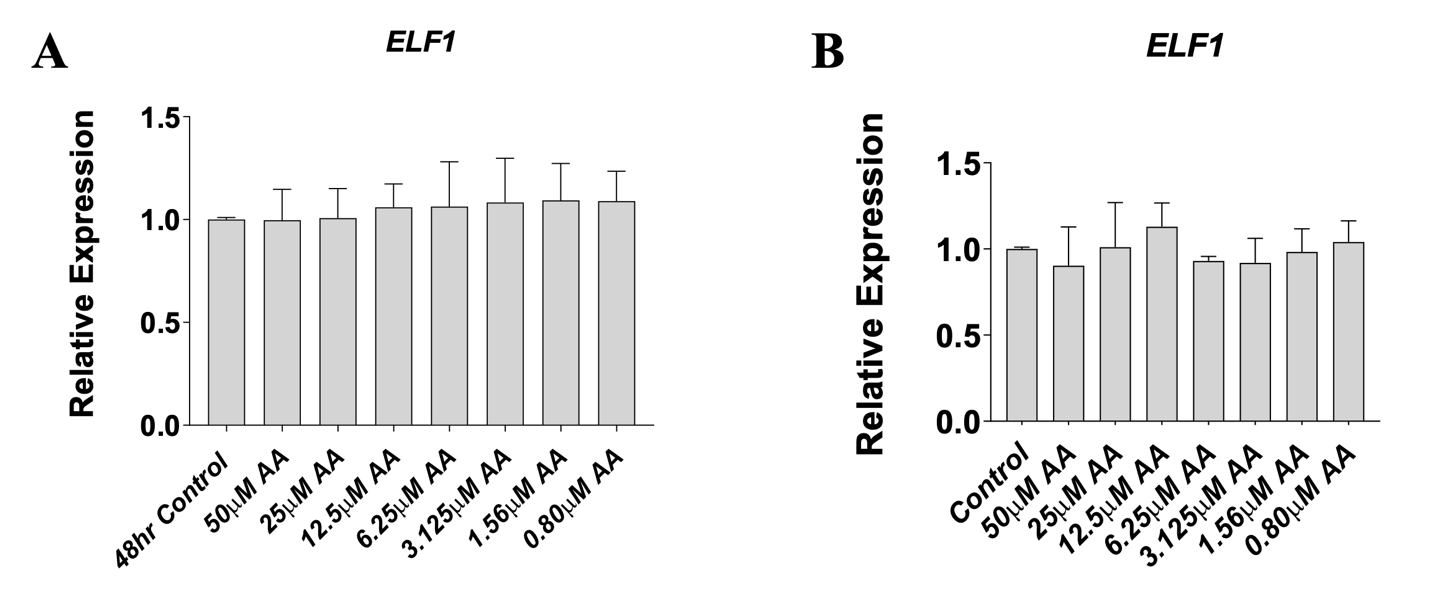
**

Supplementary Figure 3. Myoblasts (A) and Myotubes (B) were analyzed for ELF1 mRNA expression to ensure stability as a housekeeping gene. There was no significant difference between groups for myoblasts or myotubes. Data is expressed as mean ± SEM. n = 3 independent experiments.


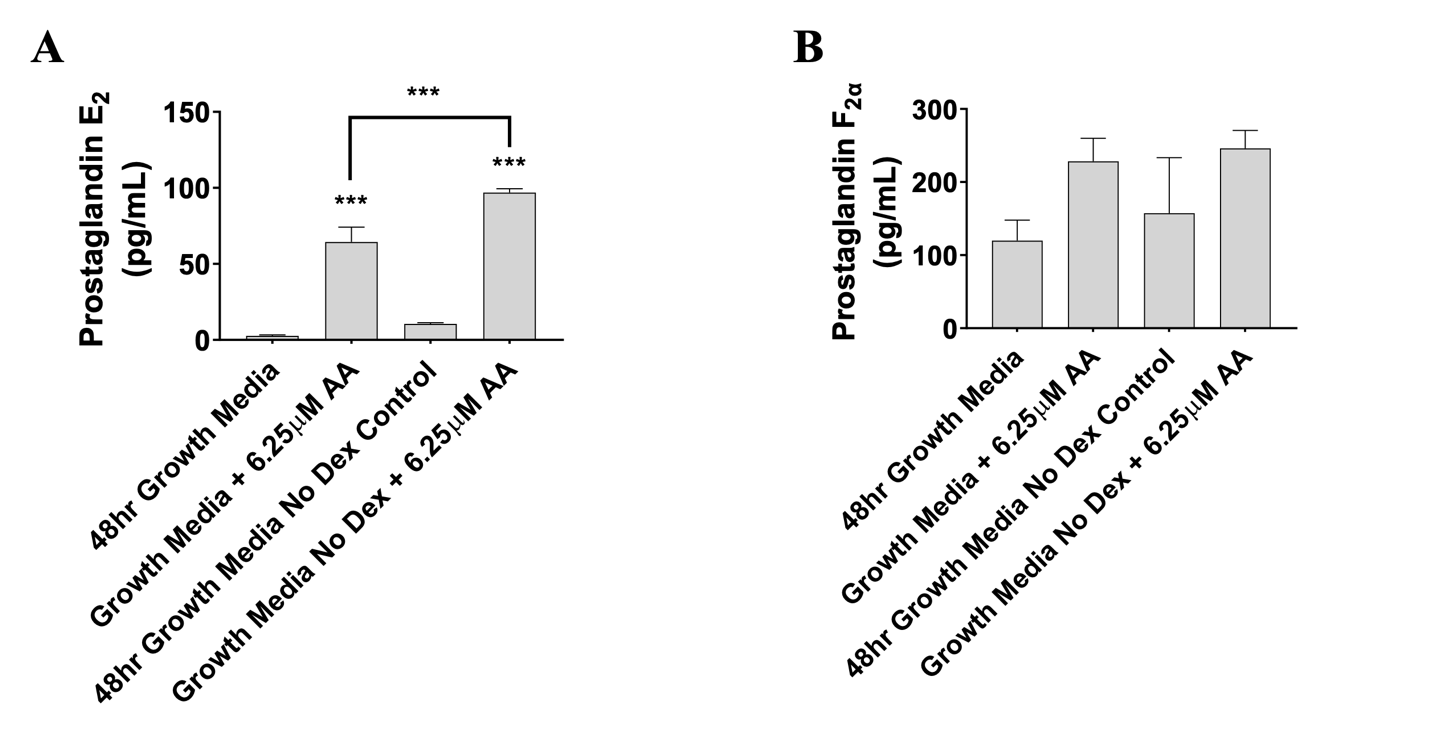


Supplementary Figure 4. Arachidonic acid stimulates prostaglandin production more without dexamethasone in the growth media. Prostaglandin hormones PGE2 and PGF2α were measured using ELISAs. (A) PGE2 and (B) PGF2α concentrations are shown for proliferating myoblasts treated with arachidonic acid with or without dexamethasone for 72 hours. Data is expressed as mean ± SEM (***p<0.001; **p<0.01; *p<0.05; all compared to respective Control).
